# FGF-23 associated with the progression of coronary artery calcification in hemodialysis patients

**DOI:** 10.1186/1471-2369-14-241

**Published:** 2013-11-01

**Authors:** Abdullah Ozkok, Cigdem Kekik, Gonca Emel Karahan, Tamer Sakaci, Alper Ozel, Abdulkadir Unsal, Alaattin Yildiz

**Affiliations:** 1 Department of Internal Medicine and Nephrology, Istanbul University, Istanbul Faculty of Medicine, Istanbul, Turkey; 2 Department of Medical Biology, Istanbul University, Istanbul Faculty of Medicine, Istanbul, Turkey; 3 Department of Internal Medicine and Nephrology, Sisli Etfal Training and Research Hospital, Istanbul, Turkey; 4 Department of Radiology, Sisli Etfal Training and Research Hospital, Istanbul, Turkey; 5 Department of Internal Medicine, Division of Nephrology, Istanbul University, Istanbul School of Medicine, 34093, Fatih Istanbul, Turkey

**Keywords:** Fibroblast growth factor-23, Coronary artery calcification, Vascular calcification, Hemodialysis

## Abstract

**Background:**

Disordered mineral metabolism is implicated in the pathogenesis of vascular calcification in hemodialysis (HD) patients. Fibroblast growth factor 23 (FGF-23) is the main regulator of phosphate metabolism. In this prospective study, we aimed to investigate the association of serum FGF-23 with progression of coronary artery calcification in HD patients.

**Methods:**

Seventy-four HD patients (36 male/38 female, mean age: 52 ± 14 years) were included. Serum FGF-23 levels were measured by ELISA. Coronary artery calcification score (CACS) was measured twice with one year interval. Patients were grouped as progressive (PG) (36 patients-48%) and non-progressive (NPG).

**Results:**

Age, serum phosphorus, baseline and first year CACS were found to be significantly higher in the PG compared to NPG group. Serum FGF-23 levels were significantly higher in PG [155 (80–468) vs 147 (82–234), p = 0.04]. Patients were divided into two groups according to baseline CACS (low group, CACS ≤ 30; high group, CACS > 30). Serum FGF-23 levels were significantly correlated with the progression of CACS (ΔCACS) in the low baseline CACS group (r = 0.51, p = 0.006), but this association was not found in high baseline CACS group (r = 0.11, p = 0.44). In logistic regression analysis for predicting the PG patients; serum FGF-23, phosphorus levels and baseline CACS were retained as significant factors in the model.

**Conclusions:**

Serum FGF-23 was found to be related to progression of CACS independent of serum phosphorus levels. FGF-23 may play a major role in the progression of vascular calcification especially at the early stages of calcification process in HD patients.

## Background

Vascular calcification is common and contributes to increased cardiovascular mortality in hemodialysis (HD) patients [1,2]. Disordered mineral metabolism and disturbances in the regulators of phosphorus homeostasis such as fibroblast growth factor 23 (FGF-23) may be implicated in the pathogenesis of vascular calcification [3,4]. FGF-23 is a novel osteocyte-derived hormone which has important roles in phosphate and vitamin D metabolism [5,6]. Studies demonstrated significant relationships between the serum FGF-23 levels and atherosclerotic burden [7], endothelial dysfunction and arterial stiffness [8] in non-uremic population. It has been reported in cross-sectional clinical studies that increased serum FGF-23 levels were associated with aortic [4], peripheral vascular [9] and coronary artery [10,11] calcifications in HD patients. High serum FGF-23 levels were also found to be independent predictors of mortality in dialysis patients [12,13]. In this prospective study, we aimed to investigate the possible effects of serum FGF-23 on the progression of coronary artery calcification in HD patients.

## Methods

Seventy-four HD patients (36 male/38 female, mean age: 52 ± 14 years) were enrolled. Mean time on dialysis was 60 ± 4 months. All patients have been receiving dialysis more than 6 months. Information on age, sex, weight, duration of HD treatment and the etiology of chronic kidney disease (CKD) was gathered by review of medical records.

All patients received thrice weekly dialysis for a 4-h period with a standard bicarbonate-containing dialysate bath, using biocompatible HD membrane (Polysulphone, FX-80 series, Fresenius, Germany). Blood flow rates ranged from 350 to 400 ml/min, while dialysate flow rate was kept constant at 500 ml/min. Adequacy of dialysis received was calculated with double pool Kt/V and Kt/V ≥ 1.4 was considered as target. All patients were on calcium acetate as phosphate binder treatment as needed according to KDOQI guidelines. Aluminum hydroxide was used as a rescue treatment for short periods. Active vitamin D treatment was also administered as calcitriol according to KDOQI guidelines. None of the patients was used selective vitamin D receptor activation treatment. One year average use of active vitamin D and phosphate binder were also calculated. Etiology of CKD included hypertension in 19 patients, diabetes mellitus in 9, chronic pyelonephritis in 6, glomerulonephritis in 6, others 11 and unknown in 23 patients.

All biochemical blood samples were collected before the midweek HD session. Laboratory values including complete blood cell counts and serum levels of urea nitrogen, creatinine, electrolytes, calcium, phosphorus, total protein, albumin, total cholesterol, triglycerides and intact parathyroid hormone (PTH) were measured. Serum phosphorus levels were measured every month. We summed up the monthly serum phosphorus levels and divided by 12 to get the one-year phosphorus mean. Intact serum FGF-23 (USCN Life Science Inc., Wuhan, China) was determined with enzyme-linked immunosorbent assay (ELISA). Intra-assay and inter-assay coefficient of variations of FGF-23 ELISA kit were reported to be <10% and <12% respectively. Sensitivity of the FGF-23 was 5.7 pg/mL.

Patients with increase of CACS more than 10% and 50 units were classified as progressive (PG) group as we described previously [14].

### Computed tomography examination

Coronary artery calcification score (CACS) was measured twice with one year interval by computed tomography. A scan run consisted of acquisition of 40 contiguous transverse two dimensional images of 3-mm-thick sections at the level above the coronary artery origins to the cardiac apex. Exposure duration was 0.1 s per tomographic level, and other parameters were 130 kVp and 630 mA. Images were acquired with electrocardiogram triggering at 71% of the R–R interval during diastole and were obtained using a 26-cm^2^ field of view and a 512 × 512 reconstruction matrix. Contrast agents were not used. A calcification was defined as a minimum of two adjacent pixels (>0.52 mm^2^) with a density over 130 Hounsfield units. The peak intensity (in Hounsfield unit) and area (in square millimeter) of the individual calcifications were calculated. As described by Agatston et al. [15], calcium scores were obtained by multiplying each area of interest by a factor indicating peak density within the individual area. Image quality and scoring accuracy was assessed by one radiologist who carefully made vessel-by-vessel and calcific focus-by-calcific focus inspections of each image. The radiologist was blinded to the clinical and laboratory results of the patients.

We divided the patients into two groups according to baseline CACS as low (CACS ≤ 30) and high (CACS >30) baseline CACS groups. We have two reason to use 30 as the cut-off value. First, since we evaluate the progression of CACS, to avoid the extreme percent increases among individuals with little or no baseline calcification, we excluded subjects with baseline calcification scores below 30 in the relative change analyses. This cut-off level was also used for this reason in the study by Chertow et al. investigating the progression of coronary calcification in HD patients [16,17]. Secondly, CACS of greater than 30 were considered clinically significant because this threshold defines the acceptable level of reproducibility in serial CT examinations [18].

Our examinations of the patients conformed to good medical and laboratory practices and the recommendations of the Declaration of Helsinki on Biomedical Research Involving Human Subjects. Written informed consent for participation in the study was obtained from all the patients. This study has been approved by Ethical Committee of Istanbul Faculty of Medicine.

#### Statistical analysis

For statistical analysis, we used the Statistical Package for Social Sciences version 16.0 (SPSS Inc., Chicago, IL). Between-group comparisons of continuous data for two groups were performed using the Student *t*-test or the Mann–Whitney *U*-test when appropriate. The *X*^*2*^ test with Yates correction and Fisher’s exact test were used for 2 × 2 contingency tables for non-numerical data, when appropriate. Correlations between numerical parameters with non-normal distribution were analyzed with Spearman’s rho correlation test. To predict the CACS progression, logistic regression analysis was performed. Results are expressed as mean ± SD unless otherwise stated. Variables with non-normal distribution were expressed as median (IQR). All tests of significance were two sided, and differences were considered statistically significant when the p-value was <0.05.

## Results

**Figure 1:**
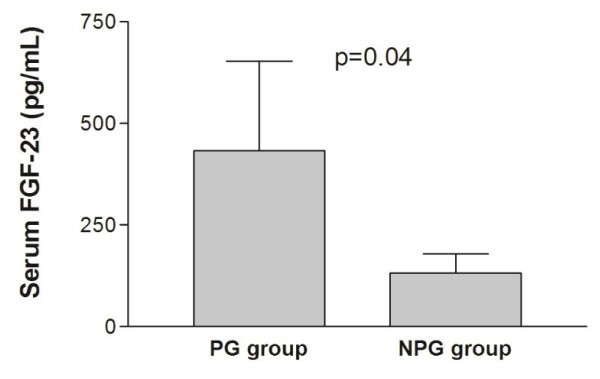
Serum FGF-23 levels were higher in PG compared to NPG group.

General characteristics and baseline biochemistry were presented in the Table 1. Comparison of PG and non-progressive (NPG) groups in terms of baseline laboratory results were given in Table 2. Accordingly; age, serum phosphorus, calcium-phosphorus product, hemoglobin, baseline and first year CACS were found to be significantly higher in the PG compared to NPG group. More importantly, serum FGF-23 levels were significantly higher in PG [155 (80–468) vs 147 (82–234), p = 0.04] (Figure 1).

**Table 1:** Baseline demographic and biochemical results of the study patients

Age (years)	52 ± 14
Time on dialysis (months)*	54 (23–96)
BMI (kg/m^2^)	24.22 ± 3.84
Kt/V	1.43 ± 0.34
Systolic BP (mmHg)	125 ± 19
Diastolic BP (mmHg)	75 ± 10
Calcium (mg/dL)	9.46 ± 0.72
Phosphorus (mg/dL)	5.44 ± 1.51
One-year phosphorus mean (mg/dL)	5.07 ± 1.19
Ca X P (mg^2^/dL^2^)	51.56 ± 15.10
Alkaline phosphatase (IU/L)*	115 (82–183)
PTH (pg/mL)*	427 (248–621)
Uric acid (mg/dL)	6.45 ± 1.21
Albumin (g/dL)	3.92 ± 0.28
C-reactive protein (mg/L)*	7.95 (2.80-19.07)
Cholesterol (mg/dL)	192 ± 51
Triglyceride (mg/dL)*	146 (103–219)
Hemoglobin (g/dL)	11.80 ± 1.44
FGF-23 (pg/mL)*	151 (82–343)
CACS (baseline)*	52 (1–767)
CACS (1^st^ year)*	120 (1–796)

(BMI: body mass index, BP: blood pressure, FGF-23: fibroblast growth factor-23, CACS: coronary artery calcification score, PTH: parathyroid hormone) *Median (IQR).

**Table 2:** Comparison results of the progressive and non-progressive groups in terms of biochemical and CACS values

	**Progressive (n = 36)**	**Non-progressive (n = 38)**	**p value**
Age (years)	56 ± 13	48 ± 14	0.02
Time on dialysis (months)*	64 (23–98)	48 (22–84)	0.23
Kt/V	1.43 ± 0.43	1.44 ± 0.48	0.82
Vitamin D dose (μg/week)*	1.50 (0–2.50)	1.20 (0.11-2.31)	0.91
Calcium (mg/dL)	9.56 ± 0.79	9.38 ± 0.64	0.29
Phosphorus (mg/dL)	5.92 ± 1.63	4.99 ± 1.25	0.008
Ca X P (mg^2^/dL^2^)	56.71 ± 16.77	46.69 ± 11.56	0.004
Alkaline phosphatase (U/L)*	116 (82–177)	115 (81–192)	0.80
PTH (pg/mL)*	468 (233–648)	401 (249–610)	0.15
Uric acid (mg/dL)	6.47 ± 1.13	6.42 ± 1.29	0.85
Albumin (g/dL)	3.87 ± 0.30	3.96 ± 0.26	0.15
C-reactive protein (mg/L)*	8.70 (2.70-19.10)	7.40 (2.80-17.60)	0.64
Triglyceride (mg/dL)*	145 (101–209)	150 (101–225)	0.20
Cholesterol (mg/dL)	190 ± 55	195 ± 47	0.69
FGF-23 (pg/mL)*	155 (80–468)	147 (82–234)	0.04
CACS (baseline)*	190 (52–935)	4 (0.30-290)	<0.001
CACS (1^st^ year)*	543 (123–1025)	1.80 (0.65-41)	<0.001

(FGF-23: fibroblast growth factor-23, CACS: coronary artery calcification score, PTH: parathyroid hormone) *Median (IQR).

Patients were also divided into two groups according to median serum FGF-23 levels. Comparison of high and low FGF-23 groups was presented in Table 3. High FGF-23 group was found to have significantly higher serum PTH and 1-year average calcitriol dose. However, serum phosphorus levels were not different between FGF-23 groups.

**Table 3:** Comparison results of the high and low FGF-23 groups in terms of biochemical and CACS values

	**HIGH FGF-23 (n = 37)**	**LOW FGF-23 (n = 37)**	**p value**
Age (years)	49 ± 14	55 ± 14	0.06
Time on dialysis (months)*	47 (22–95)	56 (35–97)	0.60
Kt/V	1.44 ± 0.44	1.42 ± 0.47	0.79
Vitamin D dose (μg/week)*	1.60 (0.70-2.93)	0.87 (0–2.02)	0.04
Calcium (mg/dL)	9.52 ± 0.59	9.41 ± 0.83	0.54
Phosphorus (mg/dL)	5.50 ± 1.49	5.38 ± 1.54	0.74
Ca X P (mg^2^/dL^2^)	52.37 ± 14.43	50.75 ± 15.90	0.65
Alkaline phosphatase (U/L)*	130 (88–216)	109 (78–174)	0.51
PTH (pg/mL)*	500 (307–702)	346 (193–549)	0.03
Uric acid (mg/dL)	6.15 ± 1.22	6.74 ± 1.15	0.03
Albumin (g/dL)	3.94 ± 0.28	3.90 ± 0.29	0.57
C-reactive protein (mg/L)*	5.50 (2.37-12.87)	12.35 (3.85-19.10)	0.68
Hemoglobin (g/dL)	11.94 ± 1.33	11.65 ± 1.56	0.40
CACS (baseline)*	47 (0.50 - 549)	68 (7–807)	0.95
CACS (1^st^ year)*	96 (1–783)	173 (3–799)	0.64
∆CACS*	18 (0–96)	2 (0–131)	0.21
% ∆CACS*	17 (0–198)	7 (0–57)	0.19

(FGF-23: fibroblast growth factor-23, CACS: coronary artery calcification score, PTH: parathyroid hormone) *Median (IQR). Δ: Delta.

**Figure 2:**
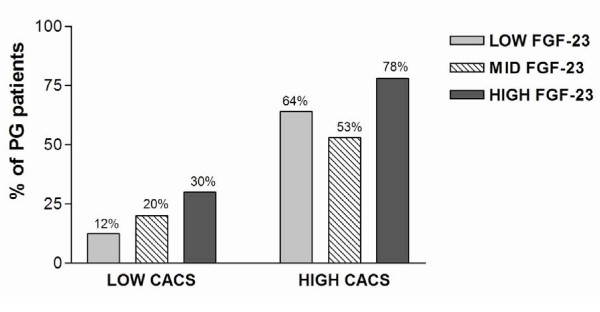
Percentages of progressed patients in different FGF-23 groups were shown in baseline low- and high- CACS groups.

**Figure 3:**
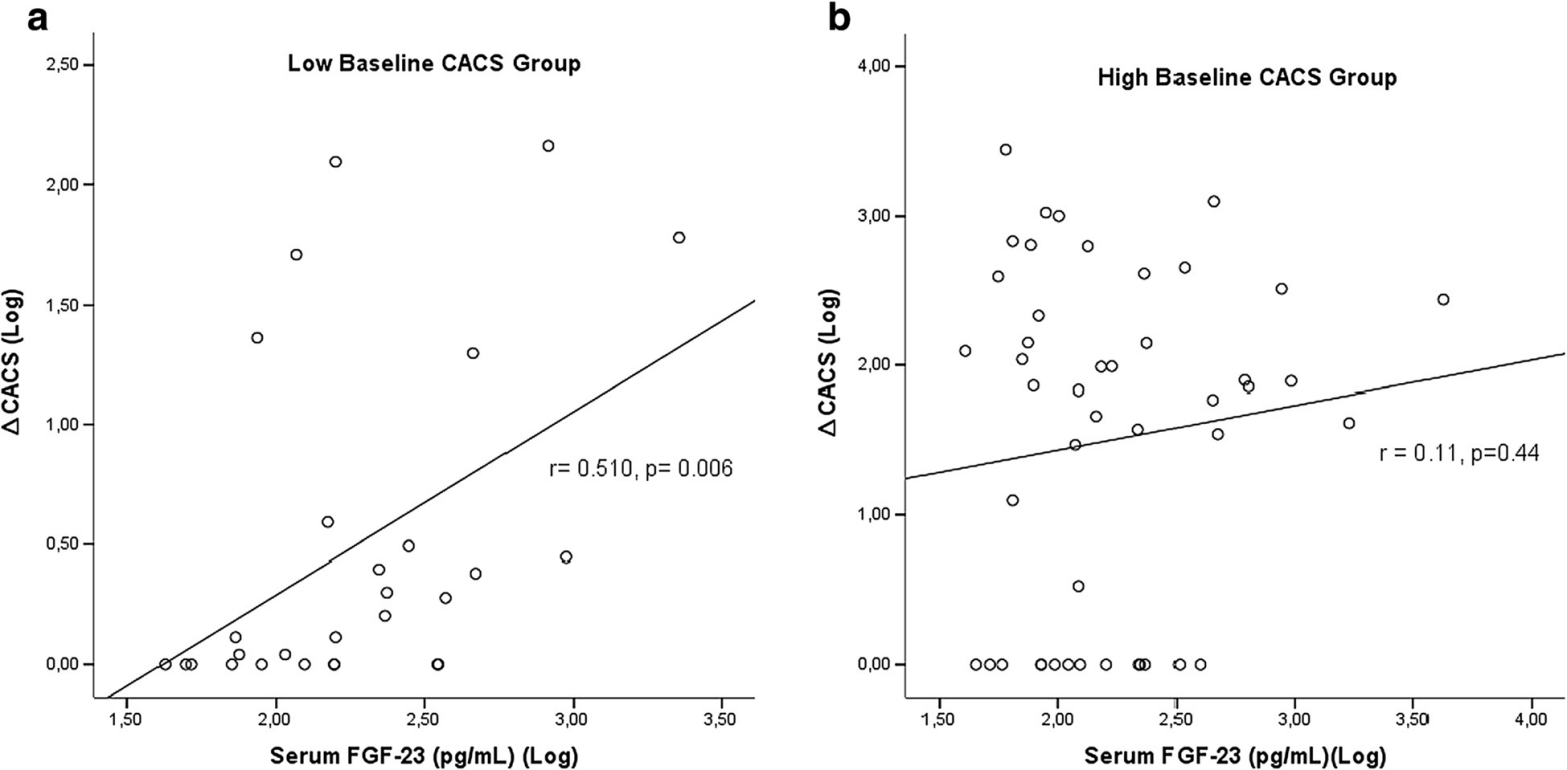
a- Serum FGF-23 levels were significantly correlated with the progression of CACS in the low baseline CACS group b- but not in high baseline CACS groups.

Serum FGF-23 levels had tendency to be related to progression of CACS (r = 0.207, p = 0.07). Patients were divided into two groups according to baseline CACS as low (CACS ≤ 30) and high (CACS >30) baseline CACS groups. Serum FGF-23 levels were similar between low and high baseline CACS groups [157 (86–351) vs 129(78–329) pg/mL, p = 0.85]. Progression of CACS (Δ CACS) in high baseline CACS group was significantly higher than the low baseline CACS group [68 (0–287) vs 0.45 (0–2.70), p < 0.001]. Confirming this finding, percent of the PG patients was higher in the high baseline CACS group compared to low baseline CACS group (30/46 (65%) vs 6/28 patients (21.4%), p <0.001), which was independent of serum FGF-23 levels. In the low baseline CACS group, percent of PG patients gradually increased with increasing serum FGF-23 levels (Figure 2). Consistent with this finding, serum FGF-23 levels were significantly correlated with the progression of CACS in the low baseline CACS group (r = 0.51, p = 0.006) (Figure 3a), however this association was not found in the high baseline CACS group (r = 0.11, p = 0.44) (Figure 3b).

Serum FGF-23 levels were significantly associated with 1-year average-serum PTH (r = 0.234, p = 0.04), uric acid (r = -0.311, p = 0.007), total cholesterol (r = 0.286, p = 0.01) and low-density lipoprotein levels (r = 0.289, p = 0.01). However, there was no correlation between serum phosphorus and FGF-23 levels (r = 0.16, p = 0.16).

In logistic regression analysis for predicting the PG patients; age, time on dialysis, serum phosphorus, baseline CACS, and FGF-23 levels were included as independent variables (-2 Log-likelihood = 68.72, p < 0.001). Serum FGF-23, phosphorus levels and baseline CACS were retained as significant factors in the model (Table 4).

**Table 4:** Model of logistic regression analysis for predicting progressive patients (-2 Log-likelihood = 68.72, p < 0.001) (FGF-23: fibroblast growth factor-23, CACS: coronary artery calcification score)

	**β value**	**95% CI for exp (β)**	**P value**
(Constant)	-7.81		0.001
Age (years)	0.040	0.99 - 1.09	0.11
Time on dialysis (months)	0.008	0.99 – 1.02	0.28
Phosphorus (mg/dL)	0.57	1.11 – 2.77	0.01
Basal CACS (Log)	0.709	1.12 – 3.66	0.01
FGF-23 (pg/ml)	0.003	1.00 – 1.007	0.03

## Discussion

In the present study, we reported that FGF-23, which is the major regulator of phosphorus metabolism in health and disease, was associated with the progression of CACS in HD patients. This association was especially true in the patients with low baseline CACS.

Serum FGF-23 increases very early in the course of CKD, long before the development of hyperphosphatemia; thus, a high FGF-23 concentration is the most sensitive and earliest marker of disordered phosphorus metabolism in CKD [19]. Furthermore, increased serum FGF-23 is regarded as one of the most important predictor of mortality in CKD even when serum phosphate levels are within normal ranges [10]. In the cross-sectional studies, it was reported that serum FGF-23 levels were associated with high atherosclerotic burden [7], endothelial dysfunction, arterial stiffness [8] and vascular calcification [4,9].

The association of serum FGF-23 levels and vascular calcification is controversial in the literature. Nasrallah et al. reported that serum FGF-23 was independently correlated with aortic calcification in non-diabetic dialysis patients [4]. In this study, serum phosphate levels did not show any correlation with calcification, suggesting an interaction between aortic calcification index and FGF-23 that was independent of serum phosphate levels. Consistent with this report, we also found that the association between serum FGF-23 levels and progression of CACS was independent of serum phosphorus levels. To our knowledge, only two cross-sectional studies were present regarding the possible relationship between serum FGF-23 and coronary artery calcification. Gutierrez et al. found significant univariate correlations between serum FGF-23 levels and CACS however the association was no longer significant after multivariable adjustment in CKD patients [10]. In a recent study performed on 16 pediatric HD patients, serum FGF-23 levels were found to be significantly associated with CACS [11]. In our prospective study, we did not observe any association between serum FGF-23 levels and baseline or first year CACS. However, FGF-23 levels were significantly higher in the PG compared to NPG group. In the very recent study by Scialla et al. [20], there was no association between serum FGF-23 levels and coronary artery calcification in the early stages of CKD patients. However the patient population of this cross-sectional study composed of only the patients with mild to moderate CKD. Patients with early CKD respond to high FGF-23 by increasing urinary phosphate excretion which results in better control of serum phosphorus, a well-known factor for development of arterial calcification. Additionally, patients with early CKD have lower FGF-23 as compared to HD patients. However, our study population composed of chronic HD patients which was completely different from that of the mentioned study. More importantly, our study is a 1-year follow-up study, and we also did not find any association between FGF-23 and CACS in the baseline period as in the study by Scialla et al. However, we have detected a significant association between serum FGF-23 and progression of CACS within 1 year. Importantly, in a very recent study by Khan et al. [21], relationship between serum FGF-23 levels and coronary calcification was investigated in 99 new to HD patients. Authors reported that serum FGF-23 was not associated with baseline CACS but serum FGF-23 levels were found to be significantly associated with CACS progression which were completely parallel to our results.

Recently, it was shown that FGF-23 might have direct effects on cardiovascular system. Faul et al. showed that intramyocardial FGF-23 injection resulted in development of left ventricular hypertrophy in experimental models suggesting the direct role of FGF-23 on heart [22]. This association was also supported by a clinical study [10]. Serum FGF-23 levels have been also reported to be related to mortality independent of serum phosphorus levels in HD patients [7] which probably represents the clinical outcome of the direct influences of FGF-23 on cardiovascular system.

An important finding of our study was the association of baseline CACS with the progression of CACS. Independent of serum FGF-23 levels, patients with high baseline CACS showed significantly more progression of CACS. The association of serum FGF-23 levels with progression of CACS was quite prominent in patients with low baseline CACS group. However this relationship was not observed in the group with high baseline CACS. These findings suggested that FGF-23 might have a major role in the early stages of vascular calcification process. In the later stages of calcification, progression of CACS could be a self-accelerating process without requiring the contribution of FGF-23. The influence of FGF-23 may also be overridden by other uremic components of calcification process in patients with higher baseline CACS.

The role of serum phosphorus in the regulation of FGF-23 secretion is not clear [23,24]. Consisting with our data, studies have failed to show any association between serum phosphate and FGF-23 levels [25,26]. Serum phosphate levels represent only a small part of total body phosphate, less than 0.1% [27]. It may be hypothesized that osteocytes sense the total body phosphate load rather than serum phosphorus levels and secrete FGF-23 accordingly.

## Conclusions

In conclusion, serum FGF-23 levels were found to be related to progression of CACS independent of serum phosphorus levels. FGF-23 may play a major role in progression of vascular calcification especially at the early stages of calcification process. Association of serum FGF-23 levels with the progression of CACS may in part explain the increased mortality rate observed in patients with high serum FGF-23 levels.

## Competing interests

The authors declare that they have no competing interests.

## Authors’ contributions

AO: Acquisition and analysis of the data, preparation of the manuscript. CK: Laboratory analysis of the parameters. GEK: Laboratory analysis of the parameters. TS: Acquisition of the data. AO: Determination and analysis of radiological data. AU: Acquisition of the data. AY: Conception and design of the study, analysis of the data, preparation of the manuscript. All authors read and approved the final manuscript.

## Pre-publication history

The pre-publication history for this paper can be accessed here:

http://www.biomedcentral.com/1471-2369/14/241/prepub
